# Iron Labeling and Pre-Clinical MRI Visualization of Therapeutic Human Neural Stem Cells in a Murine Glioma Model

**DOI:** 10.1371/journal.pone.0007218

**Published:** 2009-09-29

**Authors:** Mya S. Thu, Joseph Najbauer, Stephen E. Kendall, Ira Harutyunyan, Nicole Sangalang, Margarita Gutova, Marianne Z. Metz, Elizabeth Garcia, Richard T. Frank, Seung U. Kim, Rex A. Moats, Karen S. Aboody

**Affiliations:** 1 Department of Hematology and Hematopoietic Cell Transplantation, Beckman Research Institute, City of Hope National Medical Center, Duarte, California, United States of America; 2 Division of Molecular Medicine, Beckman Research Institute, City of Hope National Medical Center, Duarte, California, United States of America; 3 Division of Neuroscience, Beckman Research Institute, City of Hope National Medical Center, Duarte, California, United States of America; 4 Radiology MS 81, Children's Hospital of Los Angeles, Keck School of Medicine, University of Southern California, Los Angeles, California, United States of America; 5 Division of Neurology, Department of Medicine, UBC Hospital, University of British Columbia, Vancouver, British Columbia, Canada; 6 Institute for Regenerative Medicine, Gachon University Gil Hospital, Inchon, Korea; Cedars-Sinai Medical Center and University of California Los Angeles, United States of America

## Abstract

**Background:**

Treatment strategies for the highly invasive brain tumor, glioblastoma multiforme, require that cells which have invaded into the surrounding brain be specifically targeted. The inherent tumor-tropism of neural stem cells (NSCs) to primary and invasive tumor foci can be exploited to deliver therapeutics to invasive brain tumor cells in humans. Use of the strategy of converting prodrug to drug *via* therapeutic transgenes delivered by immortalized therapeutic NSC lines have shown efficacy in animal models. Thus therapeutic NSCs are being proposed for use in human brain tumor clinical trials. In the context of NSC-based therapies, MRI can be used both to non-invasively follow dynamic spatio-temporal patterns of the NSC tumor targeting allowing for the optimization of treatment strategies and to assess efficacy of the therapy. Iron-labeling of cells allows their presence to be visualized and tracked by MRI. Thus we aimed to iron-label therapeutic NSCs without affecting their cellular physiology using a method likely to gain United States Federal Drug Administration (FDA) approval.

**Methodology:**

For human use, the characteristics of therapeutic Neural Stem Cells must be clearly defined with any pertubation to the cell including iron labeling requiring reanalysis of cellular physiology. Here, we studied the effect of iron-loading of the therapeutic NSCs, with ferumoxide-protamine sulfate complex (FE-Pro) on viability, proliferation, migratory properties and transgene expression, when compared to non-labeled cells. FE-Pro labeled NSCs were imaged by MRI at tumor sites, after intracranial administration into the hemisphere contralateral to the tumor, in an orthotopic human glioma xenograft mouse model.

**Conclusion:**

FE-Pro labeled NSCs retain their proliferative status, tumor tropism, and maintain stem cell character, while allowing *in vivo* cellular MRI tracking at 7 Tesla, to monitor their real-time migration and distribution at brain tumor sites. Of significance, this work directly supports the use of FE-Pro-labeled NSCs for real-time tracking in the clinical trial under development: “A Pilot Feasibility Study of Oral 5-Fluorocytosine and Genetically modified Neural Stem Cells Expressing *Escherichia coli* Cytosine Deaminase for Treatment of Recurrent High-Grade Gliomas”.

## Introduction

Glioblastoma multiforme (GBM) is the most common primary malignant brain tumor in adults, with a mean survival of less than one year following diagnosis, despite advances in surgical, radiation and chemotherapeutic approaches [1], [2], [3]. The diffuse, highly invasive nature of glioma cells contributes to tumor recurrence, treatment failure, and lethality. New treatment modalities, including immunotherapy, gene therapy and drug delivery across the blood-brain barrier (BBB), have yet to achieve significant clinical success due to shortcomings in effectively targeting these invasive tumor cells, while minimizing toxicity to normal tissue [2]. New tumor-selective, targeted strategies are needed to achieve a significant impact on long-term survival of GBM patients.

Neural stem cells (NSCs) have inherent tumor-tropic properties to primary and invasive tumor foci, and therefore offer much promise as cellular delivery vehicles to effectively target therapeutic gene products to these invasive glioma cells [4], [5], [6]. This provides the basis for developing novel NSC-mediated treatment approaches to localize therapeutic gene products specifically to malignant primary and invasive tumor foci. Genetic modifications to NSCs by our laboratory and others have shown 70–90% therapeutic efficacy following intracranial administration of NSCs in animal models of orthotopic glioma, medulloblastoma, and melanoma brain metastases, and following intravascular administration in a mouse model of disseminated neuroblastoma [4], [6], [7], [8], [9], [10], [11]. In addition, the identification of molecular mechanisms and factors that are involved in NSC-tumor tropism have been shown in *in vitro* studies [12], [13], [14], [15], [16], [17], [18], and thus offer the further possibility of fine-tuning the tumor-selective localization of NSCs. This NSC-mediated therapeutic approach would overcome current limitations of standard therapies, while minimizing toxicity to normal tissues [19], [20].

As with any cell-based therapy, the efficacy of this treatment largely depends on the ability of the stem cells to adequately target and distribute throughout tumor sites. To maximize therapeutic benefit, optimal timing of treatment regimens must be determined according to the spatio-temporal migration of stem cells to tumor sites. We have previously quantitatively analyzed NSC-glioma distribution using 3-D modeling and mathematical algorithms. Assuming a 50 micrometer radius of action around the NSCs, this model predicts a minimum of 70–90% coverage of the primary tumor mass and invasive tumor foci [21]. However, dynamic determination of NSC migration and tumor distribution in real time is essential for optimizing treatments in pre-clinical models and designing clinical protocols. Bioluminescence and optical fluorescent imaging have been employed as non-invasive methods to track NSC migration and monitor therapeutic efficacy in animal models [22]. However, the clinical utility of these imaging modalities is limited by poor tissue penetration and low spatial resolution, making them impractical for use in patient trials. Although positron emission tomography is commonly used in pre-clinical and clinical studies for visualization of various tumors and drug interactions and for understanding tumor metabolism with high specificity, its low spatial resolution (>3–4 cm), radiation dose, and relatively short-term signal production make it a non-ideal technique for clinical tracking of cells to tumors [23], which requires extended periods of observation.

Clinical Magnetic Resonance Imaging (MRI) at 3 Tesla, however, has high spatial resolution (approximately 1 mm^3^) with excellent soft tissue contrast for non-invasive, dynamic *in vivo* assessment of cellular trafficking at multiple time points. Research on MRI cellular tracking is rapidly expanding, and many studies have been published during the last decade. Relevant studies include mouse or rat-derived stem or progenitor cells transplanted into the brain, spinal cord or vasculature of laboratory animals using strongly T1-weighted paramagnetic contrast labels such as gadolinium [24], [25], and using T2 and T2*-weighted super-paramagnetic iron oxide nanoparticles (SPIOs) [26].

## Results

### Viability and Proliferation Potential of FE-Pro Labeled NSCs

**Figure 1:**
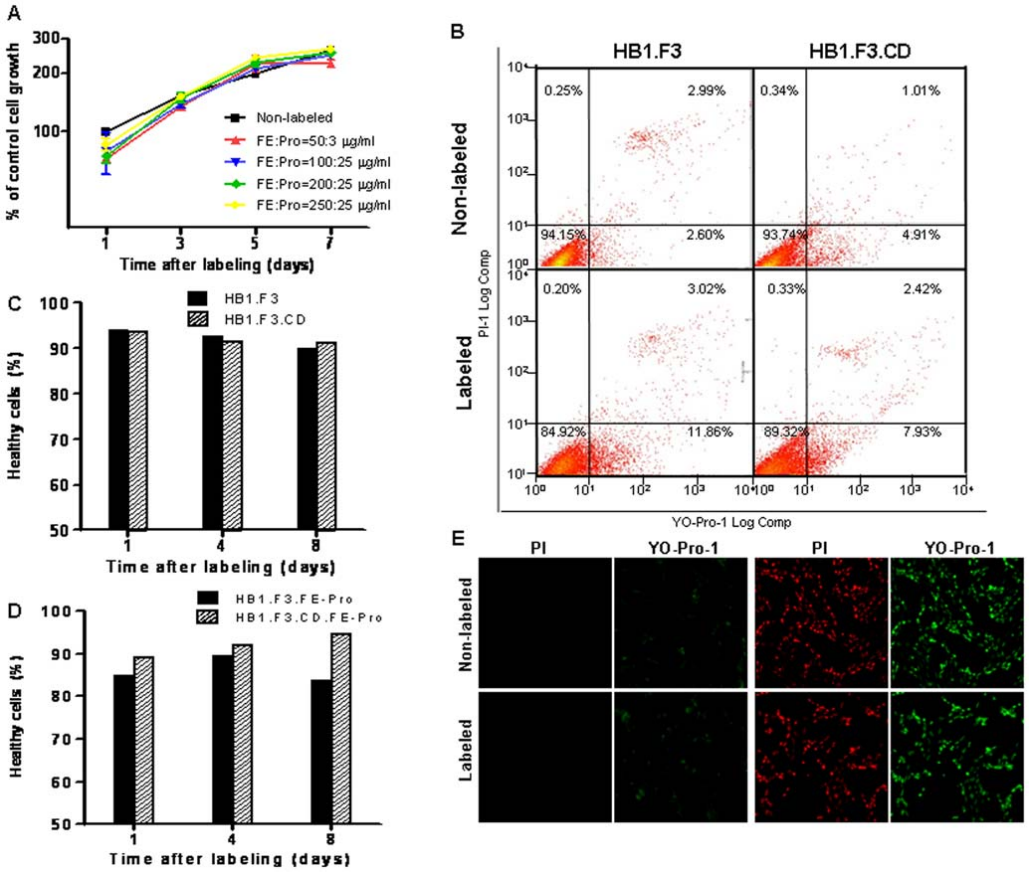
Cellular viability of FE-Pro-labeled NSCs. (A) Cellular biomass normalized to non-labeled NSC cell growth at day 1 as measured by absorbance of protein-bound sulforhodamine B (SRB) at 570 nm. Data are mean±SE of triplicate samples and were analyzed using paired t-test between non-labeled vs. each FE-Pro dosage. P<0.05 was considered statistically significant. (B) Representative FACS plots showing the viable and apoptotic cell populations at 24 hours post-label and before sub-culturing. (C–D) Bar graphs showing the percentage of healthy cells at days 1, 4 and 8 for non-labeled NSCs (C), and FE-Pro-labeled NSCs (D) after sub-culturing passage at each time point. (E): Confocal images of healthy FE-Pro labeled and non-labeled NSCs (left panel) and apoptosis-induced FE-Pro labeled and non-labeled NSCs (right panel) at Day 6 post-labeling. Staining: PI (red), YO-Pro-1 (green). A FE-Pro dosage of 50∶3 µg/ml was used for each labeled sample unless otherwise indicated. Abbreviations: FE-Pro, Ferumoxide-Protamine Sulfate complex; PI, propidium iodide; Magnification: 20×.

To determine the cellular viability and proliferation potential of FE-Pro labeled NSC lines (HB1.F3, HB1.F3.CD), we employed the sulforhodamine B (SRB) colorimetric assay and YO-Pro-1/PI to assess cytotoxicity and apoptosis, respectively. Adherent NSCs in a 96-well culture plate were labeled at various concentrations of FE-Pro complex (FE at 50, 100, 200 and 250 µg/ml, each of which was complexed with increasing Pro at 3, 10, and 25 µg/ml). The highest labeling concentration studied (FE∶Pro = 250∶25 µg/ml) was greater than 5x the dosage (FE∶Pro = 50∶3 µg/ml) for MR cellular tracking in pre-clinical and future clinical settings. We performed SRB cytotoxicity assays 1, 3, 5 and 7 days after the NSCs were labeled with FE-Pro. No significant difference in SRB staining intensity was observed between labeled and unlabeled cells (P>0.05) at each time point as measured by optical absorbance (Fig. 1A). This led us to conclude that labeling NSCs with FE-Pro did not significantly affect cell viability and proliferation over 7 days in cell culture.

To determine the plasma membrane integrity of labeled NSCs (FE∶Pro = 50∶3 µg/ml), we performed the YO-Pro-1/PI apoptosis assay on FE-Pro-labeled and non-labeled NSCs on days 1, 4, and 8 post-labeling. Flow cytometry analysis of YO-Pro-1/PI double-stained FE-Pro-labeled NSCs showed a slight (3–9%) increase in the apoptotic population compared to the non-labeled control at day 1 (Fig. 1B). However, long-term monitoring of sub-cultured labeled and non-labeled NSCs revealed similar percentages of healthy cells over time (Fig. 1C, D), indicating that labeling with FE-Pro causes no long term toxicity or effect on cellular integrity. Confocal images of both labeled and non-labeled NSCs treated with the apoptosis-inducing agent, staurosporine (STS) showed 100% cell death, whereas non-treated NSC populations reflect viabilities detected by flow cytometry (Fig. 1E). These results suggest that FE-Pro labeling has no significant effect on NSC survival (P>0.05).

### Labeling Efficiency and *In Vitro* MRI Visualization of FE-Pro-Labeled NSCs

**Figure 2:**
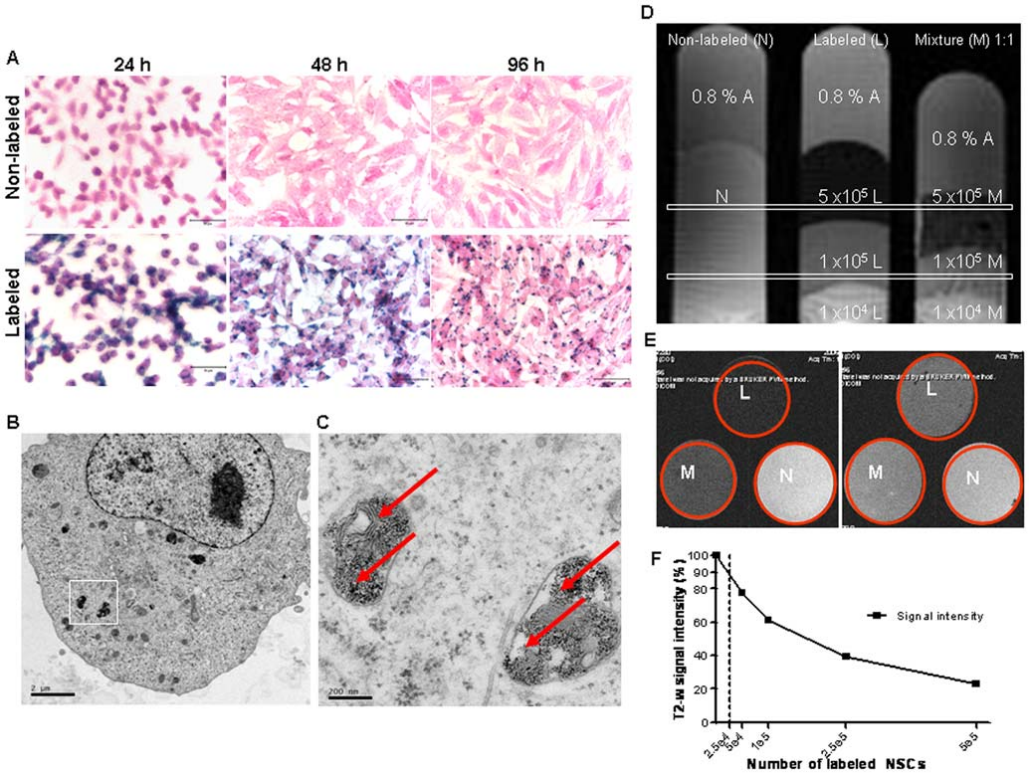
Labeling efficiency of FE-Pro. (A) Light microscopy images of Prussian blue-stained non-labeled and FE-Pro-labeled NSCs at 24, 48 and 96 hours after labeling. (B) Electron micrographs of Fe-Pro-labeled NSCs. (C) Higher magnification image of outlined area in (B). Red arrows point to internalized FE-Pro complex in membrane-bound organelles. (D–E) T2-weighted MR images of labeled (L), non-labeled (N), and an equal mixture (M) of NSCs grown in soft agar. Each phantom contained three different total numbers of NSCs (1×10^4^, 1×10^5^ and 5×10^5^) each in 500 µl of 20% DMEM and 0.8% agar. Coronal view (D) and axial view at 5×10^5^ (E. left) and 1×10^5^ (E. right) of the phantoms. Decrease in T2-w signal strength correlated with the number of labeled cells in the phantom. (F) Graph of T2-w signal intensity vs. number of labeled NSCs. Data were extracted from 5 random fields of each corresponding phantom using ImageJ and shown as mean±SE. MRI conditions: 7.0 Tesla, Gradient-Echo sequence, voxel size = 0.09 mm^3^, TR/TE = 5402.5/90 ms. Scale bars = 50 µm (A), 2 µm (B) and 200 nm (C).

To determine whether the FE-Pro label was retained by NSCs, internalized and detectable by MRI, we used Prussian blue (PB) staining, electron microscopy (EM) and *in vitro* MRI assessment, respectively. Prussian blue staining of FE-Pro-labeled NSCs indicated that greater than 95% of FE-Pro-labeled NSCs retained the iron cores over 96 h in tissue culture, with predicted dilution due to cell division (Table 1 and Fig. 2A). Unequal distribution of iron particles during cell division appeared to contribute to the small population (<5%) of PB-negative NSCs. Bright-field images of PB-stained NSCs revealed similar cell adherence and proliferation properties between FE-Pro labeled and non-labeled samples (Fig. 2A). Transmission EM micrographs showed the FE-Pro complex as an electron-dense structure in the cytoplasm of labeled NSCs (Fig. 2B). No residual extracellular FE-Pro complex was observed in the labeled samples following the final PBS/heparin wash. The iron-cores of SPIOs were localized to membrane-bound structures (red arrows; Fig. 2C), indicating that FE-Pro complexes were encapsulated rather than dispersed throughout the cytoplasm.

**Table 1:** Retention of FE-Pro label in HB1.F3.CD NSCs.

**HB1.F3.CD (Non-labeled)**	0.0±0.0 (24 h)	0.0±0.0 (48 h)	0.0±0.0 (96 h)
**HB1.F3.CD (FE-Pro-labeled)**	100.0±0.0 (24 h)	99.3±0.8 (48 h)	95.6±5.5 (96 h)

Data is displayed as means +/− SD of Prussian blue positive iron-loaded NSCs (% of total cell number). The data were obtained from 5 random fields of each independently labeled triplicate sample at 24, 48 and 96 h post-labeling.

We performed T2-weighted (T2-w) multi-spin multi-echo (MSME) MRI of FE-Pro-labeled NSCs that were suspended in an equal volume of 0.8% agarose gel and (DMEM+20% FBS) 24 h after labeling. A T_E_ of 90 ms was optimal for observing the T2-w signal gradient because it correlated with the number of labeled cells in the phantom. Longer T2 values saturated the magnetic susceptibility of the SPIOs, thereby wiping out the signals, whereas shorter T2 values did not allow optimal detection of differences in hypointense signal intensities. Equal mixtures of FE-Pro-labeled and non-labeled NSCs (1×10^5^ cells/500 µl), which mimicked one cell division, resulted in a detectable low intensity signal that could be distinguished from 100% labeled or non-labeled cells (Fig. 2D). In addition, there was an exponential relationship between T2-w signal loss and the number of FE-Pro-labeled cells (Fig. 2F). The projected 10% reduction in signal intensity visualized by MRI corresponded to 2.5×10^4^ labeled cells/500 µl. At this concentration and MRI voxel size of 0.09 mm^3^ (300 µm×300 µm×1 mm) used in this study, 9 FE-Pro labeled cells would produce detectable MRI signal reduction. The estimated number of NSCs per voxel detected in Fig. 2D and 2E was 9 FE-Pro labeled cells/voxel.

### FE-Pro-Labeled NSCs Retain Glioma-Targeting Ability and Transgene Expression *In Vitro*

**Figure 3:**
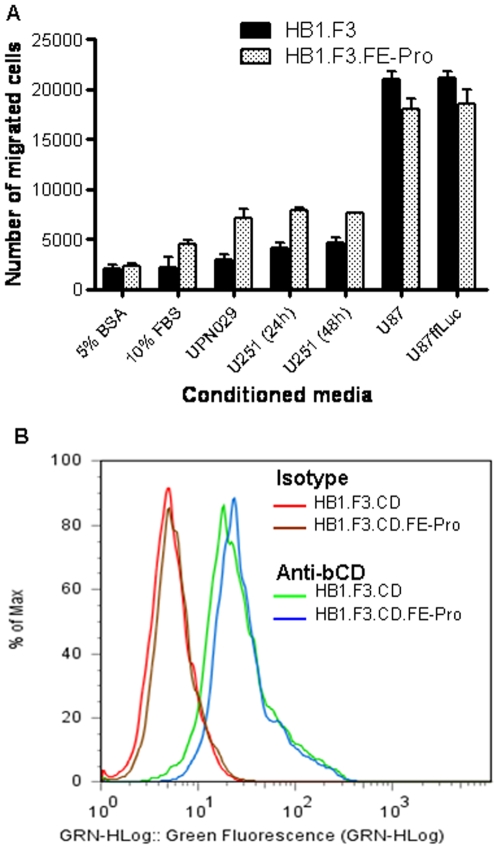
Functionality of FE-Pro labeled NSCs. (A) Results from Boyden chamber migration assays, showing inherent NSC migration towards conditioned media from U251 (media collected at 24 and 48 hours), UPN029, U87, and U87ffluc cell lines. P<0.05 was considered statistically significant. (B) Flow cytometry plot, showing expression of Cytosine Deaminase (CD) in non-labeled (red (isotype control) and green (anti-bCD)) and FE-Pro-labeled (brown (isotype control) and blue (anti-b-CD)) HB1.F3.CD cells. Abbreviations: HB1.F3.CD.FE-Pro, FE-Pro-labeled HB1.F3.CD NSCs; Anti-bCD, anti-bacterial CD primary antibody.

NSC-based tumor therapy relies upon the ability of NSCs to target tumor sites and deliver therapeutic agents to these sites. We assessed the ability of FE-Pro-labeled NSCs to target gliomas and express a therapeutic transgene (cytosine deaminase, CD) with *in vitro* Boyden chamber migration assays and immunohistochemistry, respectively. *In vitro* Boyden chamber analysis of NSC tropism to tumor chemo-attractants in conditioned media collected from serum-starved adherent tumor cells at 24 h and 48 h showed no significant difference in migratory properties between FE-Pro labeled and non-labeled NSCs (P>0.05) (Fig. 3A). To assess whether FE-Pro-labeled NSCs retained their ability to express a therapeutic transgene, we used HB1.F3 cells that carried the CD transgene (HB1.F3.CD; CD converts the prodrug 5-fluorocytosine to the anti-cancer agent 5-fluorouracil). Permeabilized HB1.F3.CD cells (±FE-Pro) were stained with antibodies against CD and analyzed by flow cytometry. More than 90% of the HB1.F3.CD cells (±FE-Pro) exhibited >10-fold increase in fluorescence intensity compared to staining with isotype-matched control antibodies (Fig. 3B). We conclude that FE-Pro labeling did not affect the expression of CD transgene (P>0.05).

### MRI Visualization of FE-Pro-Labeled NSCs Homing to Distant Tumor Site

**Figure 4:**
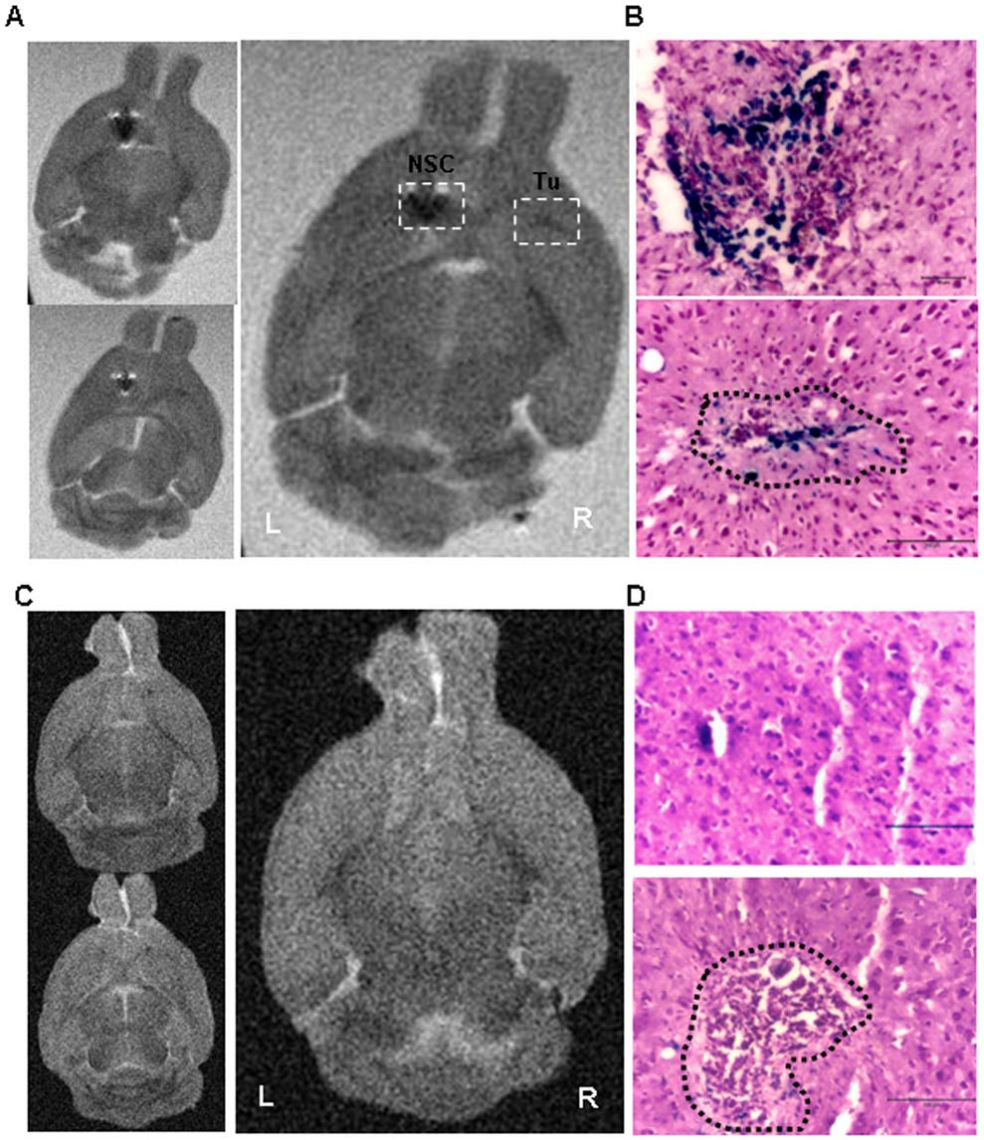
MRI Visualization of FE-Pro-labeled NSCs targeting human glioma in an orthotopic mouse model. (A) Consecutive T2-weighted MR images of mouse brain in 30% sucrose and 4% PFA. FE-Pro-labeled NSCs are shown as hypointense (dark) signals (white dotted boxes) in the left hemisphere and in the contralateral right hemisphere, where human U251 glioma cells were implanted. (B) Higher magnification, Prussian blue stained sections from the areas outlined by the boxes in (A) (top, left hemisphere; bottom, right hemisphere, tumor area outlined by black dotted line). (C) Consecutive T2-weighted MRI images of mouse brain in Fomblin that received PBS sham injection on left hemisphere and human glioma U251 on the right hemisphere. No low-intensity signals were detected in this control. (D) Higher magnification, Prussian blue stained sections from the areas outlined by the boxes in (C) (top, left hemisphere; bottom, right hemisphere, tumor area outlined by black dotted line). MRI conditions: 7.0 Tesla, Rapid Acquisition Relaxation Enhancement sequence, 78 µm/pixel, 300 µm/slice, T_R_/T_E_ = 1500/23.1 ms. Scale bars = 100 µm (B and D).

**Figure 5:**
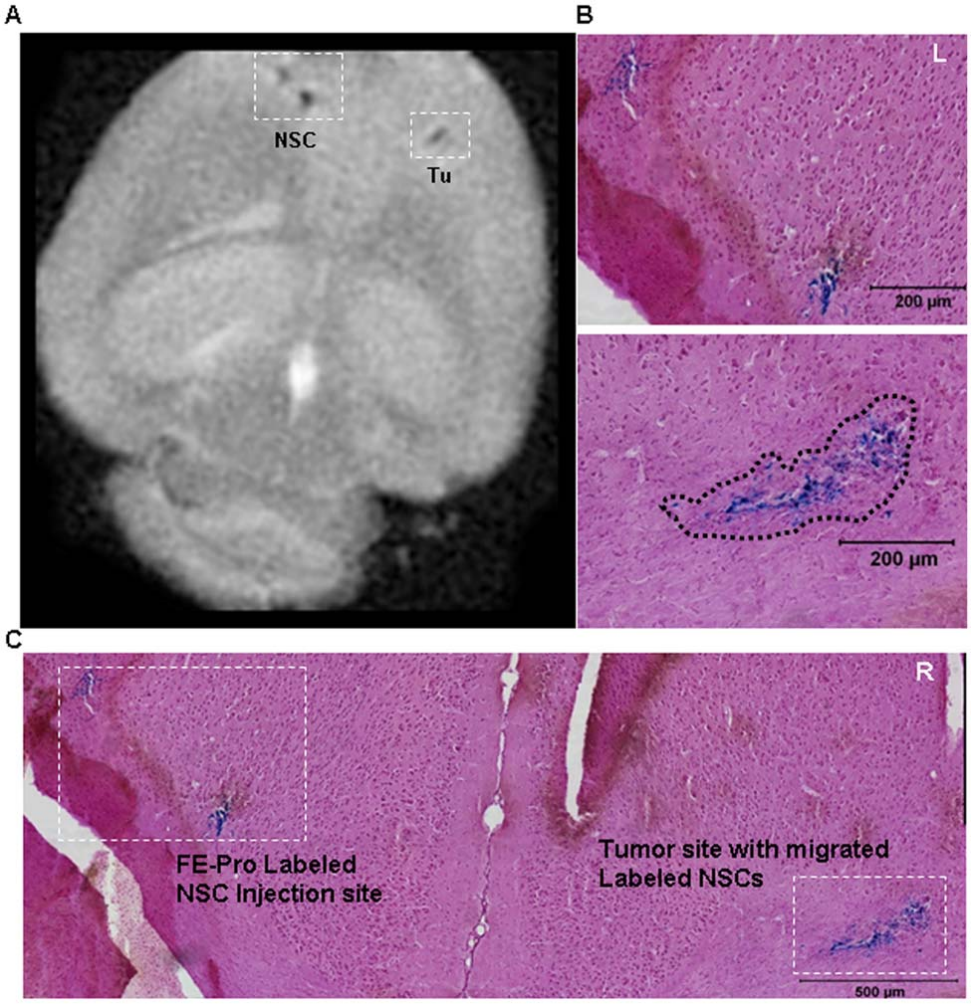
Sensitivity of MRI monitoring of FE-Pro-labeled NSCs targeting human glioma. (A) T2-weighted MR image of mouse brain in Fomblin, showing two distinct signal voids generated by FE-Pro-labeled NSCs that were injected ∼200 µm apart from each other on the left hemisphere and a hypointense signal generated by FE-Pro-labeled NSCs that migrated to the contralateral tumor site (white dotted boxes). Approximately 600 FE-Pro-labeled NSCs constituted a detectable signal void. (B and C) Prussian blue stained section from the region shown in (A). Higher magnification images (B, tumor area denoted by black dotted line) of the regions outlined in (C), showing PB-positive labeled NSCs corresponding to the hypointense signal sites in (A). MRI conditions: 7.0 Tesla, Rapid Acquisition Relaxation Enhancement sequence, 78 µm/pixel, 300 µm/slice, T_R_/T_E_ = 1500/23.1 ms. Scale bars = 200 µm (B), 500 µm (C).

To establish the feasibility of using MRI to visualize homing of NSCs to the tumor site, brains were removed from mice four days after intracranial administration of FE-Pro-labeled NSCs (total of 14 days after contralateral U251 glioma implantation), and were analyzed using *ex vivo* MRI. Low intensity T2-weighted signal was observed at the NSC injection site, as well as at the tumor site, that was distinguishable from native low signal in the surrounding tissue (Fig. 4A and Fig. 5A). Injection of FE-Pro-labeled HB1.F3 cells (1.0×10^4^ to 2.5×10^5^ cells) resulted in equally detectable hypointense MRI signals at the contralateral tumor site. Post-MRI histological analysis with Prussian blue staining confirmed the presence of iron-labeled NSCs at the tumor site (Fig. 4B, 5B and 5C), correlating with the hypointense MRI signals (Fig. 4A and 5A). These data suggest that relatively low numbers of FE-Pro-labeled NSCs can be tracked by MRI for migration and distribution. The ability of MRI to perform high resolution imaging of NSC sites was demonstrated by injecting two doses of 5000 FE-Pro-labeled NSCs, 500 µm apart. Two distinct signal voids were observed (Fig. 5A). These hypointense signals corresponded to the spatial distribution of the PB-positive iron-labeled cells (Fig. 5A and 5B). A fraction of the FE-Pro-labeled NSCs migrated to and infiltrated the tumor site in all contralateral samples (Fig. 4A, 5A and 5B). The intensity of the T2-w signal at the NSC injection site and tumor site appeared to correlate to the density of PB-positive labeled NSCs at these sites (Fig. 5A and 5B). The estimated number of FE-Pro-labeled NSCs extracted from representative brain sections that gave rise to detectable T2-w signal loss in 300-µm thick MRI slices was as few as 600 NSCs (please see Materials and Methods). Sham injection (PBS), contralateral to the tumor implants (Fig. 4C) resulted in no hypointense signal in MRI images and correlated with a lack of PB staining in these histological samples (Fig. 4D). Because the tumors were very small (∼200–500 µm), which mimicked residual glioma foci, the tumors themselves did not yield detectable MRI signal.

### The FE-Pro Complex is Retained within NSCs after Implantation into Mouse Brain

**Figure 6:**
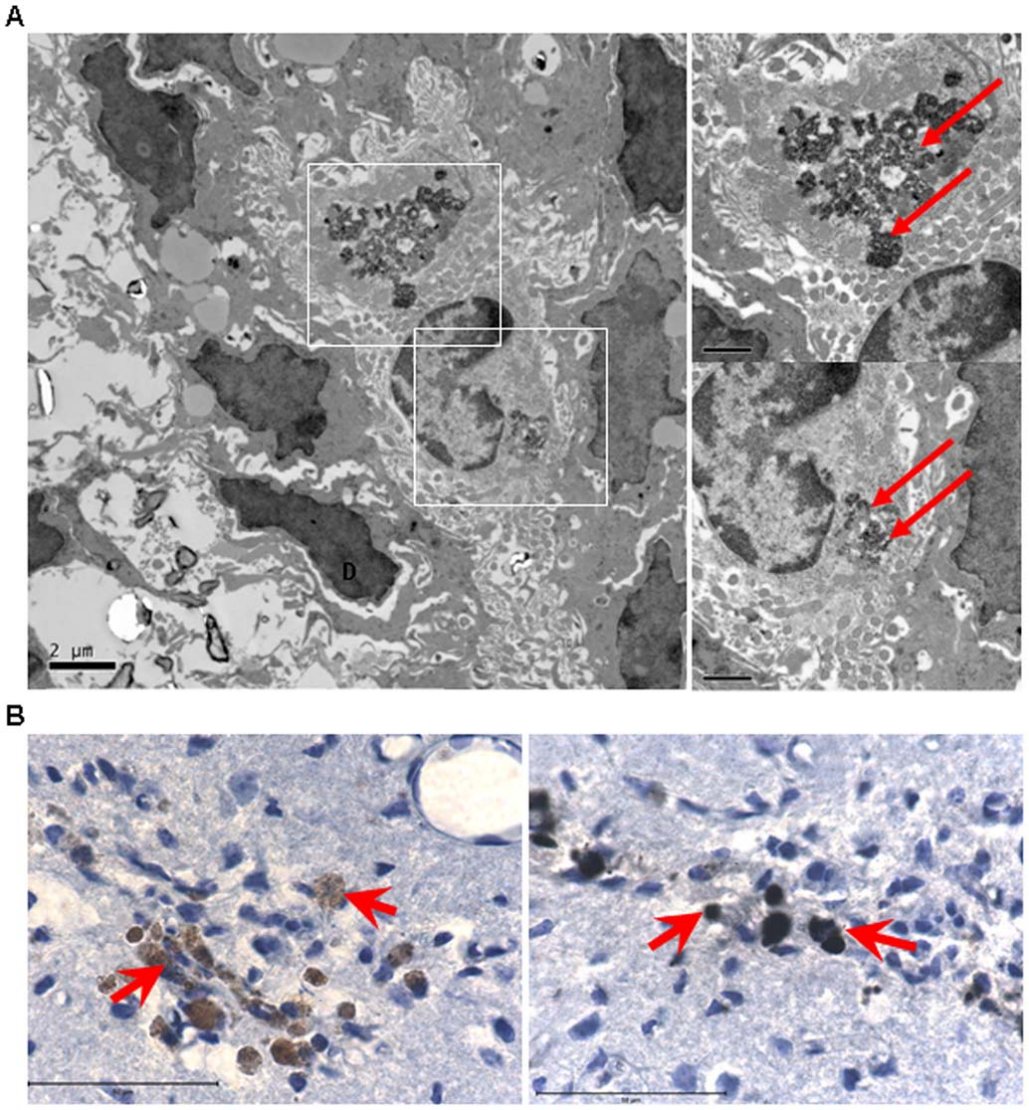
Retention of FE-Pro complex within labeled NSCs after implantation in tumor-bearing mice. (A) Electron micrograph of mouse brain that received intra-tumoral NSC administration (left). On the right are higher magnification images of the outlined areas in (A) (top and bottom). The FE-Pro complex is shown as an electron-dense iron label within the cells (solid boxes, A; red arrows, B). This is comparable to the morphology of encapsulated iron label in labeled NSCs *in vitro* (Fig. 2B). (B) Light microscopy images of huMito-positive cells (migrated FE-Pro-labeled NSCs and U251 cells) at the tumor site (B left, red arrows), and huMito and Prussian blue double-stained FE-Pro-labeled NSCs in parallel sections (B right, red arrows). Scale bars = 2 µm (A left), 1 µm (A right) and 50 µm (B).

To determine if the FE-Pro complexes were well-retained and encapsulated within NSCs after implantation into the mouse brain and subsequent tumor-tropic migration, the brain samples were analyzed with TEM and immunohistochemistry. In TEM micrographs of brain tissue from mice that received an intratumoral injection of FE-Pro-labeled NSCs, we observed electron-dense FE-Pro complexes that were still internalized within the membrane-bound structures, demonstrating that the complexes were retained for at least 5 days post-labeling (4 days post-implantation) (Fig. 6A). The morphology of iron-containing NSCs 5 days post-labeling was identical to the *in vitro* FE-Pro-labeled NSCs. Immunohistochemical analysis with human-mitochondrial antibody (huMito) was performed to identify the human NSCs, followed by PB staining to detect iron-positive cells in the previously described brain sample shown in Fig. 4A and 4B. Co-localization of huMito and PB staining was observed, indicating that iron complexes were in human cells and not engulfed by mouse macrophages or dispersed in extracellular space (Fig. 6B).

## Discussion

Glioblastoma is a highly invasive tumor with recurrence rate of 98%, which is in most cases rapidly fatal [27], [28], [29]. The failure of current clinical and experimental therapies to eradicate disseminated glioma cells results in tumor recurrence and a median survival of 3–6 months [2]. Furthermore, currently available imaging technologies are not sufficiently sensitive for detection of small tumor satellites, which are responsible for recurrence [2]. An emerging therapeutic platform using NSCs for targeted delivery of anti-cancer drugs specifically to tumor sites should allow higher therapeutic index in glioma patients, as exemplified in pre-clinical animal models of solid tumors [4], [6], [7], [8], [9], [10], [11]. However, in order to effectively use NSC-mediated cancer therapy, the migration of NSCs to disseminated tumor sites, as well as the therapeutic response, must be assessed. Although *in vivo* MRI for monitoring cellular trafficking has been extensively studied in pre-clinical animal models of regenerative medicine, few studies have used such an MR imaging modality in stem cell-mediated tumor therapy [30], [31]. Therefore, we sought to further advance a cellular tracking methodology using MRI for monitoring NSC migration and tumor targeting in an orthotopic glioma xenograft model.

NSCs labeled with FE-Pro displayed normal cellular proliferation and viability, when compared to non-labeled NSCs. Although we detected a slight increase in apoptosis (7–8%) at 24 h after FE-Pro labeling, the proportion of apoptotic cells in FE-Pro-labeled NSCs decreased to a level comparable to the non-labeled control NSCs (5%) at later stages of NSC culture (up to 9 days). Other studies have reported effects of FE-Pro labeling comparable to ours; however, such analyses were performed only up to 4 days in culture [41]. Our data are consistent with findings reported for iron-labeled human hematopoietic and mesenchymal stem cells in studies of tumor angiogenesis [32], [33], [34], and neurosphere-derived NSCs in mouse models of stroke [35], [36]. Using electron microscopy, we detected iron nanoparticles in the cytoplasmic compartment, enclosed within endosomes, suggesting that these particles are biologically inactive and cause little or no interference with cell physiology. Studies have shown that these nanoparticles ultimately enter the normal iron metabolic pathways [37], [38], [39].

The feasibility of cell-based therapies depends upon the ability of transplanted cells to maintain their intended therapeutic functions *in vivo.* It has been demonstrated that iron-labeled neural progenitor cells, mesenchymal stem cells, and hematopoietic stem cells retain their regenerative and therapeutic potential *in vivo* [32], [33], [34], [35], [36], [40], [41], [42], [43]; however, it has been reported that Feridex complexed with poly-L-lysine blocked the differentiation of human MSCs into chondrocytes [44]. Of note, the success of our cancer therapeutic approach requires that FE-Pro-labeled NSCs retain their tumor-targeting ability and expression of the therapeutic transgene, but does not necessitate differentiation or long-term intra-parenchymal integration of FE-Pro-labeled NSCs. Further, our previous results with solid tumor models showed that NSCs are self-eliminated during the course of anti-cancer therapy [7], [8], [11]. Using Boyden chamber cell migration assays, we demonstrated that FE-Pro-labeled NSCs migrate to tumor-conditioned media *in vitro*, whereas our *ex vivo* MRI demonstrated NSC tumor-targeting in glioma-bearing mice. In addition, FE-Pro-labeled HB1.F3.CD cells maintained expression of the transgene expression *in vitro* as shown by immunocytochemistry and flow cytometry analyses. *In vivo* ^19^F MR Spectroscopy can be used to determine expression of CD transgene in patients by measuring the conversion of 5-fluorocytosine prodrug into 5-fluorouracil during a therapeutic regimen.

A concern with using SPIO labeling for MRI cellular tracking is dilution of the contrast agent due to cellular division and the consequent decrease in the ability to detect the signal by MRI. Indeed, SPIO-labeled cells show a gradual reduction in intracellular iron particles with increased post-labeling incubation time. This reduction has been attributed to cell division and/or exocytosis [45], [46]. Additionally, signal dilution can also occur as a result of biodegradation and entry of iron into metabolic pathways [45], [47]. Our data show that >99% of NSCs were labeled with FE-Pro up to 48 h post-labeling, and that >95% of FE-Pro-labeled NSCs retained Prussian blue-positive iron nanoparticles at 96 h post-labeling. The amount of iron appeared to be diluted, as expected, due to cell division. Furthermore, Neri *et al.* reported 80% labeling efficiency of NSCs with Resovist alone or Resovist complexed with poly-L-lysine *in vitro* [48], thus, our FE-Pro labeling protocol shows superior iron labeling efficiency of NSCs.

To establish feasibility of MRI for visualization of FE-Pro labeled NSCs, it was important to determine the *in vitro* detection level by MRI. We were able to detect 9 FE-Pro-labeled NSCs per 300 µm×300 µm×1 mm size voxel. Furthermore, a difference in T2-weighted signal reduction was observed between the 100% labeled NSCs and non-labeled NSCs, as well as in an equal mixture of labeled and non-labeled NSCs. This shows that after one cycle of cell division, we are still able to detect the FE-Pro-labeled NSCs. Significantly, our previous studies show that NSCs do not divide *in vivo*, when implanted into the brain of glioma-bearing mice (Aboody et al, unpublished results). These findings suggest that FE-Pro-labeled NSCs will retain the iron nanoparticles to a sufficient degree to produce hypo-intense signal, contrast-enhanced from surrounding tissue in T2-weighted MRI. However, due to heterogeneity in the structure and composition of brain tissue and differences in MRI sequence and parameters, it is difficult to directly extrapolate the exact number of labeled cells *in vivo* based on quantitative assessment from *in vitro* studies. We verified in *ex vivo* MRI that FE-Pro labeled NSCs injected contra-lateral to frontal lobe tumor in the mouse brain, migrated along white matter tracts to the tumor site and were detected 4 days after administration of NSCs as hypointense signal. Studies of CD34-positive hematopoietic stem cell incorporation into tumor neovasculature and of neural progenitor cell migration to sites of spinal cord injury showed that such labeled stem cells were visible by MRI up to 1–2 weeks after their administration [33], [49], [50], [51], [52]. In addition, transplanted SPIO-labeled pancreatic islets in patients have been tracked by MRI for up to 6 months post-transplantation [39]. We intend to further verify the successful use of FE-Pro labeling of NSCs for *in vivo* MRI cellular tracking in pre-clinical animal models for the planned clinical trial in glioma patients.

In addition to internalization and retention of the FE-Pro label by NSCs, it is important to establish the sensitivity of MRI FE-Pro-labeled NSCs at a particular resolution with MRI sequence field strength. It has been reported that even a single cell can be tracked in MRI in case of highly phagocytic, large cells, such as macrophages [53]. Because iron nanoparticles produce hypointense signals by causing magnetic susceptibility in surrounding water molecules, the labeled cells can cause a ‘blooming effect’ in an area of interest due to iron overload [31]. In our studies, similar numbers of NSCs migrated to tumor sites within 4 days after administration, independent of the dosage of cells implanted. A total of 10,000 NSCs administered to the left frontal lobe at two sites 500 µm apart appeared in MRI as distinct hypointense signals. A fraction of NSCs that migrated to the tumor implant in the opposite hemisphere were visible at 600 FE-Pro-labeled NSCs per site with an MRI resolution of 76 µm×76 µm×300 µm.

Qualitative analysis of the spatio-temporal targeting of glioma by NSCs in MRI requires further confirmation that the detected MRI signal arose from FE-Pro-labeled NSCs. A concern is the possible false positive interpretation of the MRI signal, which may be produced by macrophages that have engulfed non-viable labeled NSCs or freely-dispersed iron nanoparticles in the brain tissue [54]. Although 10–20% of macrophages, when mixed with stem cells, took up iron in *in vitro* Boyden chamber assays [54], Arbab et al. found no co-localization of mouse macrophages and iron-containing (Prussian blue-positive) areas of brain tissue [33], [34]. Here, our TEM data confirmed that the iron label was encapsulated within endosomes *in vivo* after migration and/or distribution at the tumor sites. Histological analysis revealed co-localization of anti-human mitochondrial antibody and Prussian blue staining, suggesting that the iron-containing cells are of human origin. Although tumor cells may take up iron nanoparticles released from non-viable NSCs, the enclosure of iron in endosomes, as evidenced in our TEM data, suggest this process is unlikely. Further support for tumor-specific targeting comes from our data showing no identifiable presence of iron-labeled NSCs in non-tumor-containing healthy brain tissues. Likewise earlier reports have shown that NSCs, inoculated into non-tumor-bearing brains of laboratory animals, did not randomly dissipate into adjacent normal tissue or to the contra-lateral hemisphere [55].

The study of NSC trafficking in glioma therapy will help us define the optimal time-frame for NSC delivery, the dose of anti-cancer therapeutic, dosing regimen, and route of administration. Our MRI cell tracking methodology will also aid the interpretation of data obtained in clinical trials. In combination with standard MRI techniques (e.g., gadolinium-enhanced MRI, diffusion-weighted MRI, or MR Spectroscopy), our method will allow us to better assess disease activity as related to the presence of therapeutic NSCs.

## Materials and Methods

### Human Neural Stem Cells

The HB1.F3 human neural stem cell line was established by retroviral transduction of v-*myc* into primary human neural stem cells isolated from fetal telencephalon of 15 weeks gestation [56]. This parental HB1.F3 cell line was further transduced retrovirally to stably express the *E. coli* cytosine deaminase gene (CD; EC 3.5.4.1), designated as HB1.F3.CD NSCs. Both the HB1.F3 and HB1.F3.CD lines are well-characterized and multipotent, non-tumorigenic and non-immunogenic [10], [56], [57], [58]. NSCs were cultured as an adherent monolayer in Dulbecco's Modified Eagle's Medium (DMEM) (Invitrogen, Carlsbad, CA) containing high glucose (4.5 g/l), 1 mM sodium pyruvate, 2 mM L-glutamine, 100 µg/ml streptomycin and 100 units/ml penicillin, supplemented with 10% fetal bovine serum (FBS) in 6% CO_2_ at 37°C. (Note: the HB1.F3.CD cell line has been approved for clinical use by the NIH Recombinant DNA Advisory Committee).

### FE-Pro Complex and Labeling of NSCs

NSCs were labeled with FE-Pro as described by Arbab *et al*. [59] with some modifications. Briefly, Ferumoxide (FE) (Feridex IV, Berlex Laboratories, Wayne, NJ) with a total iron content of 11.2 mg/ml was diluted to the desired concentration (100–500 µg/ml) in serum-free DMEM. Protamine Sulfate (Pro) solution (1 mg/ml) was freshly prepared in distilled water from a 10 mg/ml stock solution (AM Pharm Partner, Schaumburg, IL), and added to the serum-free DMEM-FE solution to achieve 2X the desired final FE-Pro concentration. The solution was agitated for 1 min to allow the FE-Pro complex to form and was immediately added to adherent NSCs (80% confluence). Following incubation for 2 h at 37°C, 6% CO_2_, an equal volume of DMEM/10% FBS was then added to the NSCs. After 24 h of further incubation, the NSCs were washed three times in sterile PBS, and one time in PBS containing 10 U/ml heparin (Abraxis, Schaumburg, IL) to remove the residual FE-Pro from the cell surface, followed by a rinse in PBS and re-suspension in DMEM/10% FBS for *in vitro* experiments or sterile PBS for intracranial injections into glioma-bearing mice.

### Analysis of Cellular Viability

The sulforhodamine B (SRB) cytotoxicity assay was used to assess the viability of FE-Pro-labeled NSCs [60]. Briefly, the samples (plated in a 96-well culture plate) were fixed with ice-cold 10% trichloroacetic acid at 4°C for 1 h, stained with 100 µl of 0.4% SRB in 1% acetic acid per well for 15 min at room temperature (RT), rinsed 3 to 4 times with 1% acetic acid, and solubilized in 100 µl of 10 mM Tris-base. The intensity of SRB staining was evaluated using a Spectra Max 250 Microplate Spectrophotometer at 570 nm. For early and late apoptosis analysis, NSCs were labeled, trypsinized and re-suspended at 1×10^6^ cells/ml and stained with 1 µM YO-Pro-1 (Invitrogen) for 20 min and 1.5 µM propidium iodide (PI) (Invitrogen) for 5 min. NSCs were re-plated for further study time points and the same procedures repeated. The dye uptake was analyzed using flow cytometry. The flow cytometry data were confirmed with confocal imaging using a Zeiss LSM 510 confocal microscope (Carl Zeiss Microimaging Inc., Thornwood, NY). As a positive control, 1 h staurosporine (100 nM) treatment was used as an apoptosis-inducing agent.

### Labeling Efficiency Studies

Labeling efficiency studies were performed with iron-sensitive Prussian blue (PB) staining, electron microscopy and magnetic resonance imaging. NSCs were cultured in 6-well culture plates to 80% confluence, labeled with FE-Pro for 24 h, and stained with PB at 24, 48 and 96 h post-FE-Pro-labeling. Briefly, NSCs were fixed with 4% paraformaldehyde (PFA) for 5–10 min, washed with distilled H_2_O (dH_2_O) and incubated in freshly prepared equal volumes of 4% w/v K_4_Fe(CN)_6_ and 1.2 mM HCl (Sigma-Aldrich, St. Louis, MO) for 30 min at RT, followed by a rinse with dH_2_O and counterstained with nuclear fast red (Sigma-Aldrich, St. Louis, MO). Bright-field micrographs of PB-stained, FE-Pro labeled and unlabeled samples were taken and the percentages of PB-positive versus -negative NSCs were calculated from 5 random fields. For MRI analysis of labeling efficiency, see MRI section below.

### Expression of the Cytosine Deaminase Transgene

Adherent NSCs were labeled with FE-Pro, as described above, and re-suspended at 5×10^6^ cells/ml in staining/wash buffer (SWB) (94% PBS [without Ca^2+^ and Mg^2+^], 5% FBS and 0.001% w/v NaN_3._ NSCs were fixed and permeabilized (Fix and Perm kit, Caltag), rinsed and immunostained with anti-bCD primary antibody (0.01 µg/ml; BD Pharmingen, San Diego, CA) or mouse IgG1 kappa isotype control antibody (0.01 µg/ml; BD Pharmingen) for 20 min at RT. The samples were then washed twice with SWB, stained with 10 µg/ml of goat anti-mouse-IgG/IgM-FITC (BD Pharmingen) and incubated for 20 min at RT in the dark. After two final rinses with SWB, the cell pellet was re-suspended to 2.5×10^4^ cells/µl in SWB. The number of CD-positive cells was analyzed by flow cytometry (Guava Technologies, Hayward, CA).

### Cell Migration Assay

*In vitro* cell migration assays were performed using 96-well cell culture plates with polycarbonate inserts (Millipore, Billerica, MA) with 8-µm pore diameter. Tumor cell-conditioned media were prepared by addition of serum-free media (SFM) to adherent tumor cells (∼75% confluence), followed by incubation at 37°C, 6% CO_2_ for 24 or 48 hours. 5% BSA/DMEM, 10% FBS/DMEM and conditioned media from tumor cell lines were added to the lower chamber of 96-well plates (150 µl/well, triplicate samples). Inserts were placed into wells and suspensions of labeled and non-labeled NSCs were added in the upper chamber (3×10^4^ cells/100 µl suspended in 5% BSA/DMEM to each well). After incubation for 4 hours at 37°C, the cells that did not migrate were removed from the inner surface of the filter. The membrane tray was then placed in a new lower chamber containing pre-warmed detachment buffer (Accutase, Sigma-Aldrich, St. Louis, MO) at 37°C for 10 min. Detached cells in the buffer were then transferred to a V-bottom 96 well plate and centrifuged at 1500 rpm for 5 min, buffer aspirated and froze at −20°C overnight. The data were analyzed using the CyQuant Green according to the manufacturer's recommended protocol. Standard curve was generated using known number of labeled and non-labeled cells and their respective fluorescence intensities (triplicate samples).

### Human Glioma Xenografts

Nude/nude mice (Charles River) were anesthetized with an intra-peritoneal injection of 132 mg/kg Ketamine and 8.8 mg/kg Xylazine. Animals were then immobilized in a stereotactic apparatus and received stereotactically-guided intracranial injections of U251 human glioma cells (5×10^4^ cells/2 µl sterile PBS) into the right frontal lobe (2 mm lateral, 0.5 mm anterior to bregma, tracked from a depth of 2.5 mm to 2.25 mm to 2.0 mm; 0.667 µl of cell suspension was injected at each level for optimal tumor/host brain contact and engraftment). Tumor cell injections were performed with a 30-gauge 5-µl Hamilton syringe over 3–5 minutes. After retracting the needle over 2–4 minutes, bone-wax was used to occlude the burr hole, Betadine was applied to the surgical area, and skin was closed with skin glue or sutures. Buprenorphine analgesic was administered intra-peritoneally at 0.05 mg/kg to relieve post-operative pain. Tumor implants grew to the size of ∼0.2–0.5 mm by Day 10, mimicking small, residual tumor foci. All animal protocols were approved by the City of Hope and/or Children's Hospital, Los Angeles IACUC. When mice appeared to be in discomfort or distress as judged by independent animal care personnel with no knowledge of the protocol design, animals were euthanized.

### Administration of FE-Pro Labeled NSCs

FE-Pro labeled HB1.F3 and HB1.F3.CD NSCs were detached by trypsinization and centrifuged for 5 min at 1200 rpm. The supernatant was discarded and NSCs were resuspended in sterile PBS (1×10^4^, 1×10^5^, or 2.5×10^5^ cells/2 µl). On day 10 post-tumor injection, the NSCs were injected intratumorally and contralaterally at similar coordinates of injection site of tumor cells. Prior to NSC injections, the mice were anesthetized, immobilized in a stereotactic frame. FE-Pro labeled NSCs, non-labeled NSCs, or PBS alone was then administered as described above.

### In Vitro and Ex Vivo MRI

For *in vitro* MRI analysis, equal volumes of different concentrations (1×10^4^ cells/500 µl, 1×10^5^ cells/500 µl, 5×10^5^ cells/500 µl) of non-labeled, FE-Pro-labeled and an equal mixture of labeled and non-labeled NSCs were suspended in 0.8% w/v agarose gel and visualized using a 7.0 Tesla Bruker Pharmascan with Paravision 4.0 software at the Children's Hospital of Los Angeles Small Animal Imaging Core. Images were reconstructed using Bruker Paravision 4.0. T2-weighted MRI was performed with a multi-spin multi-echo (MSME) sequence of T_R_ = 5300 ms and 10 echos (T_E_ = 15–150 ms). The matrix size was 256×256 with field of view (FOV) of 3×3 cm. Average (binning) was 1 with slice thickness of 1 mm. The T2-weighted signal strength as correlated to the number of labeled cells was then obtained from 5 random fields of each corresponding phantom using NIH Image J and displayed as percent signal loss versus number of labeled cells.

For *ex vivo* MRI analysis, mice were euthanized at day 14 (4 days post-NSC injection) and perfused with PBS, followed by 4% PFA. Brains were harvested and post-fixed in 4% PFA supplemented with 30% (w/v) sucrose. MR imaging of *ex vivo* brain samples was conducted using a 19 mm horizontal magnetic bore in a 7.0 Tesla Bruker Pharmascan with Paravision 4.0. MR images were reconstructed using the standard Paravision software, and exported in DICOM format to E-film 1.8.2 for visualization and conversion to 2-D tiff files. During imaging, harvested brains were immersed in Fomblin, an MRI negative agent, to reduce the background noise. T2-weighted rapid acquisition with relaxation enhancement was used with average ranging for 4–24 for image quality, slice thickness of 300 µm, FOV of 2×2 cm, T_R_/T_E_ = 1500–2000/23.1–33.3 ms. In-plane resolution was 76 µm/pixel. For estimation of the number of FePro-labeled cells detectable by *ex vivo* MRI in mouse brain, we collected thirty 10-µm thick sections from the brain, and randomly selected 6 sections that spanned 300 µm of distance corresponding to MRI image in Fig. 5A. Prussian blue-positive NSCs were counted in these 6 sections, and the total number of NSCs within the 300-micrometer thick brain tissue was extrapolated. Immunohistochemical analysis with human-mitochondrial antibody (huMito) (Chemicon, MAB 1273) was performed to identify the human NSCs as previously described [7], and double-stained with PB to visualize iron-positive cells.

### Electron Microscopy

Internalization of the FE-Pro complex was visualized with electron microscopy using standard techniques. FE-Pro-labeled NSC pellets (fixed in 1.5% glutaraldehyde in 0.1 M cacodylate buffer) or harvested brains (post-fixed in 4% PFA supplemented with 30% sucrose) were processed with standard electron microscopy techniques and imaged with an FEI Tecnai G2 transmission electron microscope (TEM).

### Statistical Analysis

The following statistical analyses were used: two-tailed Student's t-test and ANOVA. See figure legends for specific details. GraphPad Prism and/or Excel were used to determine the statistical significance. The data are shown as mean±SD or mean±SEM. P<0.05 was considered statistically significant.
